# Flexible Piezoresistive Tactile Sensor Based on Polymeric Nanocomposites with Grid-Type Microstructure

**DOI:** 10.3390/mi12040452

**Published:** 2021-04-16

**Authors:** Da-Huei Lee, Cheng-Hsin Chuang, Muhammad Omar Shaikh, Yong-Syuan Dai, Shao-Yu Wang, Zhi-Hong Wen, Chung-Kun Yen, Chien-Feng Liao, Cheng-Tang Pan

**Affiliations:** 1 Department of Electronic Engineering, Southern Taiwan University of Science and Technology, Tainan City 71005, Taiwan; dhlee@stust.edu.tw; 2 Institute of Medical Science and Technology, National Sun Yat-sen University, Kaohsiung 80424, Taiwan; chchuang@imst.nsysu.edu.tw; 3 Sustainability Science and Engineering Program, Tunghai University, Taichung City 407224, Taiwan; omar.offgridsolutions@gmail.com; 4 Department of Mechanical and Electro-Mechanical Engineering, National Sun Yat-sen University, Kaohsiung City 80424, Taiwan; shannondai@mem.nsysu.edu.tw (Y.-S.D.); sywang@mem.nsysu.edu.tw (S.-Y.W.); 5 Department of Marine Biotechnology and Resources, National Sun Yat-sen University, Kaohsiung City 80424, Taiwan; wzh@mail.nsysu.edu.tw; 6 Department of Mechanical and Automation Engineering, I-Shou University, Kaohsiung City 84001, Taiwan; alden0113@gmail.com; 7 Department of Emergency Medicine, Kaohsiung Armed Forces General Hospital, Kaohsiung City 80284, Taiwan

**Keywords:** tactile sensors, piezoresistivity, polymer nanocomposites, multi-wall carbon nanotubes (MWCNTs), polydimethylsiloxane (PDMS)

## Abstract

Piezoresistive tactile sensors made using nanocomposite polymeric materials have been shown to possess good flexibility, electrical performance, and sensitivity. However, the sensing performance, especially in the low-pressure range, can be significantly improved by enabling uniform dispersion of the filler material and utilization of effective structural designs that improve the tactile sensing performance. In this study, a novel flexible piezoresistive tactile sensor with a grid-type microstructure was fabricated using polymer composites comprising multi-walled carbon nanotubes (MWCNTs) as the conductive filler and polydimethylsiloxane (PDMS) as the polymeric matrix. The research focused on improving the tactile sensor performance by enabling uniform dispersion of filler material and optimizing sensor design and structure. The doping weight ratio of MWCNTs in PDMS varied from 1 wt.% to 10 wt.% using the same grid structure-sensing layer (line width, line spacing, and thickness of 1 mm). The sensor with a 7 wt.% doping ratio had the most stable performance, with an observed sensitivity of 6.821 kPa^−1^ in the lower pressure range of 10–20 kPa and 0.029 kPa^−1^ in the saturation range of 30–200 kPa. Furthermore, the dimensions of the grid structure were optimized and the relationship between grid structure, sensitivity, and sensing range was correlated. The equation between pressure and resistance output was derived to validate the principle of piezoresistance. For the grid structure, dimensions with line width, line spacing, and thickness of 1, 1, and 0.5 mm were shown to have the most stable and improved response. The observed sensitivity was 0.2704 kPa^−1^ in the lower pressure range of 50–130 kPa and 0.0968 kPa^−1^ in the saturation range of 140–200 kPa. The piezoresistive response, which was mainly related to the quantum tunneling effect, can be optimized based on the dopant concentration and the grid microstructure. Furthermore, the tactile sensor showed a repeatable response, and the accuracy was not affected by temperature changes in the range of 10 to 40 °C and humidity variations from 50 to 80%. The maximum error fluctuation was about 5.6% with a response delay time of about 1.6 ms when cyclic loading tests were performed under a normal force of 1 N for 10,200 cycles. Consequently, the proposed tactile sensor shows practical feasibility for a wide range of wearable technologies and robotic applications such as touch detection and grasping.

## 1. Introduction

Human society has entered the information age in the 21st century. Sensing technology is the primary source of information technology and the front–end basis for obtaining data. Aside from representing the functions of the five human senses, sensors can also detect physical quantities that cannot be felt by the human sense. The application of sensor technology covers various fields, such as industrial automation, transportation, military, aerospace, disaster forecasting, medical, health care, etc. According to the available statistical data, the global sensor market is experiencing rapid growth, which is forecasted to grow as sensors lie at the heart of enabling technologies such as Internet–of–Things (IoT), robotics, big data, and artificial intelligence. Sensor development has gradually become a global focus, especially in the United States, Japan, and Germany. Today, the level of sensing technology has become an important indicator of whether a country’s industrial infrastructure is developed [1,2,3].

Based on the application need, a range of sensors including optical, tactile, and sonic sensors have been developed utilizing novel transducer mechanisms and materials. For robotic applications, tactile sensors are instrumental since they provide information that cannot be accessed via remote optical sensors such as cameras and lidars. Additionally, the ability to touch and feel is critical in unstructured and harsh environments and can help the robot manipulate objects more effectively. In addition, tactile sensors have been developed and used in wearable devices, consumer electronics, mechanical exoskeletons, and a range of other applications in recent years. The idea of artificial electronic skin has also garnered research interest for aiding the robot detect important variables such as touch, force applied, slippage and temperature, and pressure mapping on the surface [4,5,6,7].

Among the different transduction mechanisms utilized in tactile sensors such as piezoelectric, capacitive, optical, ultrasonic, and triboelectric, piezoresistive tactile sensors have advantages, which include a wide choice of novel sensing materials such as hybrid organic–inorganic nanocomposites and nanowire assemblies, high sensitivity, excellent flexibility, planar device design and ease of integration with simple electronic readout modules [8]. In particular, piezoresistive tactile sensors made by using nanocomposite polymeric materials have been shown to possess improved flexibility, electrical performance, sensitivity, chemical stability, and biocompatibility. Furthermore, the progress in scalable nanomaterial production has led to the development of a range of flexible resistive tactile sensors that primarily utilize nanomaterial filler–polymer matrix composites [9,10,11]. Since nanomaterials can agglomerate in uncured viscous polymers due to Van der Waals forces, it can lead to issues such as performance deviation and low repeatability. In addition, the concentration of the filler nanomaterial also significantly affects sensor performance, while lack of effective sensor structure can lead to damping effects and reduced sensitivity, especially in the low–pressure range [12,13,14,15].

**Figure 1:**
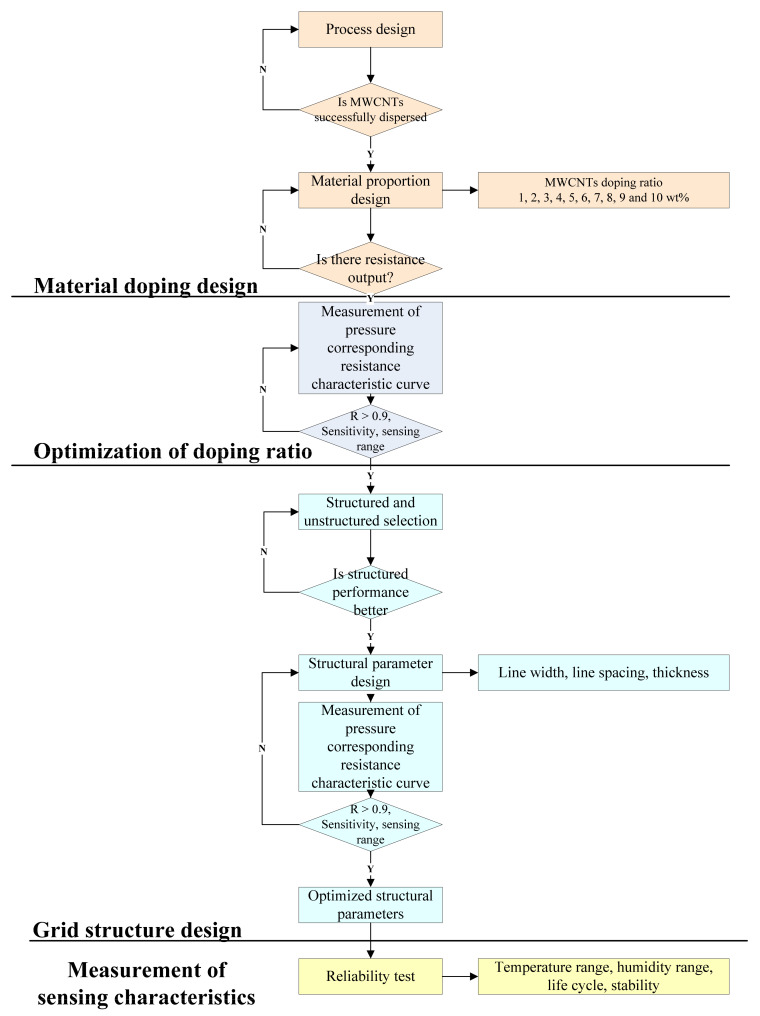
Flow diagram to highlight the research process of the proposed tactile sensor.

In this study, a novel flexible piezoresistive tactile sensor with a grid–type structure was fabricated using polymer composites comprised of multi–walled carbon nanotubes (MWCNTs) as the conductive filler and polydimethylsiloxane (PDMS) as the polymeric matrix. The research focused on improving the tactile sensor performance, especially in the low–pressure sensing range, by enabling uniform dispersion of filler material, optimizing MWCNT doping concentration, and improving sensor design and structure [16,17]. The proposed tactile sensor demonstrated a repeatable response with high sensitivity observed in the low–pressure range (0–30 KPa) and could be potentially utilized for a wide range of applications. The Flow diagram of the research is shown in Figure 1.

## 2. Design and Fabrication

### 2.1. Sensor Design

**Figure 2:**
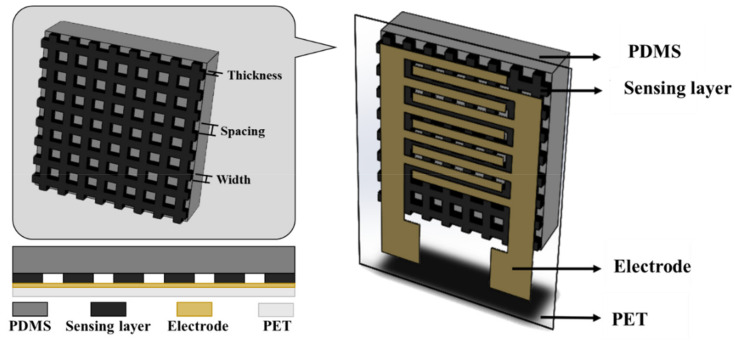
Schematic of the flexible piezoresistive tactile sensor with grid–type microstructures.

The flexible piezoresistive tactile sensor consists of a wear–resistant silver interdigitated electrode printed on polyethylene terephthalate (PET), a grid–structured sensing layer, and a PDMS protective layer, as shown in the schematic in Figure 2. PDMS displays good reproducibility for the strain behavior of repetitive structures, which makes it a suitable material for soft sensors. The sensitivity and sensing range of the sensor with different doping ratios and grid structures were discussed in this study. The grid–structured sensing layer was formed and optimized using dimensional parameters that include line width, line spacing, and thickness. The grid structure design was made using AutoCAD, followed by laser carving in which a polymethyl methacrylate (PMMA) substrate was ablated by CO_2_ laser to complete the grid–structured sensing layer mold. Next, the MWCNT/PDMS was poured into the mold for curing to form the sensing layer. A PDMS coating was used as a protective layer and also aids in releasing the sensing layer from the PMMA mold. The sensing layer was then integrated with the PET–based interdigitated (IDT) electrodes to obtain the final sensor. The use of simple techniques such as laser carving/cutting and screen printing enabled us to fabricate these sensors without the use of cleanroom facilities, high vacuum–based deposition techniques, and photolithography for patterning, which greatly reduced the time and cost.

### 2.2. Sensing Principle

**Figure 3:**
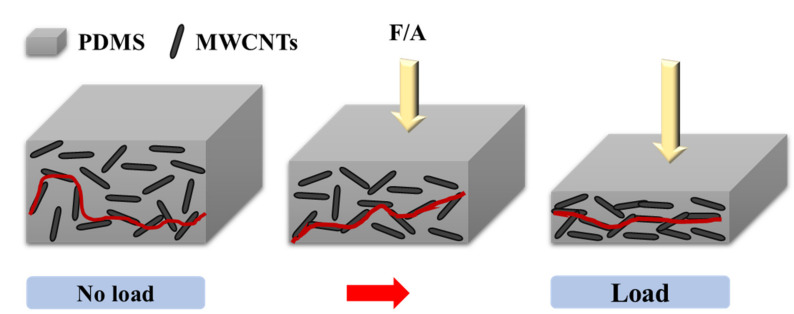
Schematic illustration of the sensor operation principle.

A piezoresistive type tactile sensor was utilized in this study. Unlike passive piezoelectric sensors, which generate electrical signals when mechanically stimulated, piezoresistive sensors are active sensors and need to be supplied with an external voltage. The resistance that the current experiences while flowing through our polymeric nanocomposite under an external voltage depends on the ability of the MWCNT to provide a conductive pathway via the electron tunneling mechanism. As pressure is applied to the sensor surface, the polymeric gap between MWCNTs decreases, which increases the tunneling effect and consequently reduces the observed resistance. However, the doping concentration of the MWCNT is critical to ensure an optimized percolation threshold and enable a piezoresistive response that is repeatable and sensitive in the required pressure sensing range [18,19,20]. The schematic illustration of the sensor operation principle is shown in Figure 3.

### 2.3. Sensing Material Preparation and Experimental Methods

**Figure 4:**
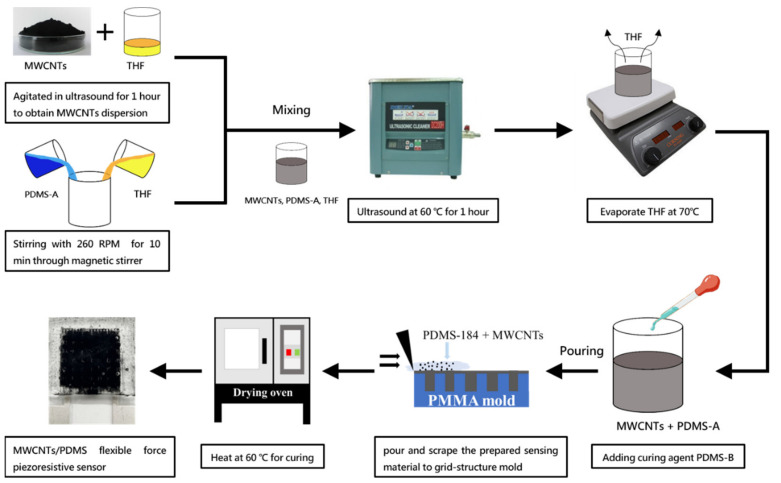
Schematic of the process flow diagram to fabricate the sensing material.

The presence of electrostatic and Van der Waals forces between MWCNTs causes them to easily agglomerate together, thus making it difficult to achieve homogeneous dispersion, which in turn affects the electrical performance of composite material. Hence, it was necessary to disperse MWCNTs effectively and uniformly into the PDMS. Adding MWCNTs directly into the PDMS resulted in difficulties in subsequent stirring and dispersion. Therefore, first dispersing MWCNTs in tetrahydrofuran (THF), followed by mixing with PDMS, was observed to promote uniform dispersion. An optimized amount of THF is crucial to ensure MWCNT dispersion, while too high an amount prolongs evaporation time and creates defects such as cracks and holes in the composite after curing. The experimental process is schematically illustrated in Figure 4.

Material Preparation–MWCNTs were added to 2.5 g of THF and ultrasonicated for 60 min to ensure uniform dispersion. Then, 2.5 g PDMS–A was added, and the resulting mixture was ultrasonicated at 60 °C for 60 min, followed by THF evaporation at a constant temperature of 70 °C. After evaporating the THF, 0.25 g of PDMS–B was added and stirred for 5 min. Finally, the composite was vacuumed for 30 min to remove any trapped air bubbles.

MWCNT Doping Ratio–In this study, 10 different MWCNT/PDMS doping composite ratios were utilized in which the MWCNT content was varied from 1 wt.% to 10 wt.%. Using a fixed grid–structured sensing layer (line width, line spacing, and thickness = 1 mm), the optimized doping ratio, which resulted in improved sensitivity in the pressure range of 0–200 kPa, was studied. The constant parameters used in the design of composites with different MWCNT doping ratios are shown in Table 1.

### 2.4. Sensor Fabrication

**Figure 5:**
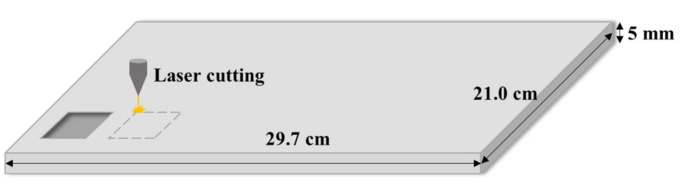
Schematic of the laser processing method to fabricate the mold.

The sensing layer was fabricated using an acrylic mold (thickness of 5 mm, a length of 29.7 cm, and a width of 21.0 cm), which was patterned using a laser processing method, as shown in Figure 5. First, AutoCAD was used to pattern grid structures with different dimensions that were then carved onto the acrylic substrate by laser ablation. There were two main factors affecting the laser carving process—laser power and speed. Therefore, by controlling the processing parameters, a grid structure of different depths can be obtained. Setting a higher laser power or lower speed will result in a deeper groove structure. The optimized parameters for laser carving included a laser power of 60% and a speed of 60 mm/s. Finally, the patterned acrylic mold was ultrasonicated for 3 min to remove any surface residues, followed by drying in an oven at 80 °C for 5 min.

Due to the precision limit of the laser carving machine, the minimum achievable resolution was 0.5 mm. The three parameters for the grid structure design used in this study were line width, line spacing, and thickness. Experiments 1 to 5 used line spacing as the variable, experiments 6 to 10 used thickness as the variable, and experiments 11 to 15 used line width as the variable. The grid structure design parameters are shown in Table 2.

After the acrylic mold fabrication, the MWCNT/PDMS sensing layer material was poured into the mold, followed by scraping the excess and performing vacuum treatment at a pressure of 680 mmHg for 30 min to remove trapped air bubbles. Next, a baking step was performed at 100 °C for 60 min, followed by spin coating a PDMS protective layer using a PDMS A: B ratio of 10:1. The same steps of vacuum treatment and baking were performed, followed by demolding to obtain the finished sensing layer. Finally, the sensing layer was integrated with a wear–resistant silver paste–based interdigitated electrodes (IDT) printed on flexible PET substrates to obtain the completed piezoresistive tactile sensor.

### 2.5. Measurement System

**Figure 6:**
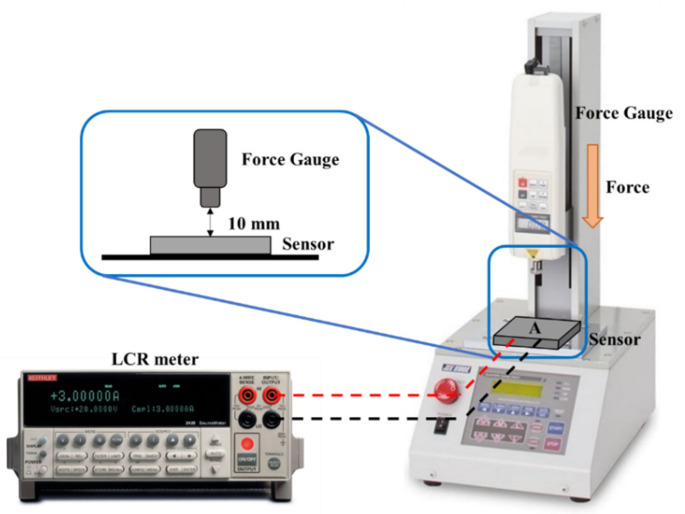
Schematic of the static normal force measurement platform.

Static Normal Force Measurement Platform—The developed tactile sensor used a force gauge integrated into an automatic vertical servo stand machine (ALGOL Instrument Co., LTD., Taoyuan City, Taiwan) to apply a normal force to the sensor surface and an LCR meter to measure its resistance response. Compared with manual testing, automatic machine testing can avoid errors caused by human inconsistencies, nonvertical angles, improper force clamping, and insufficient precision during pressing and extension. The static normal force measurement platform used in this study is schematically illustrated in Figure 6. The sensor was placed on the stage, and a normal applied force was exerted and measured by the force gauge that has an acrylic disc with a diameter of 8 mm and a thickness of 2 mm attached to the surface. The measured force range was 0–10 N, which corresponds to a pressure range of 0–200 kPa, and measurements were made at every force interval of 0.5 N (10 kPa). Each measurement was performed three times and sustained for 10 s to ensure a stable sensor response without being affected by environmental factors.

Linear Conversion of Voltage Signal Output–Since the proposed tactile sensor has a variable piezoresistive output that is inversely proportional to the static normal force, impedance matching can be connected in series with the sensor through a voltage divider circuit to convert the signal. A linear output was observed and the mathematical expression used for converting the signal into the output voltage *V_out_* is shown in Equation (1), where *R*_1_ is the variable resistance output of the sensor, *R*_2_ is the impedance matching connected to the voltage divider circuit, and *V_in_* is the DC voltage source input of 5 V [21,22].

(1) $$V_{out}=\frac{R_{2}}{R_{1}+R_{2}}V_{in}$$

**Figure 7:**
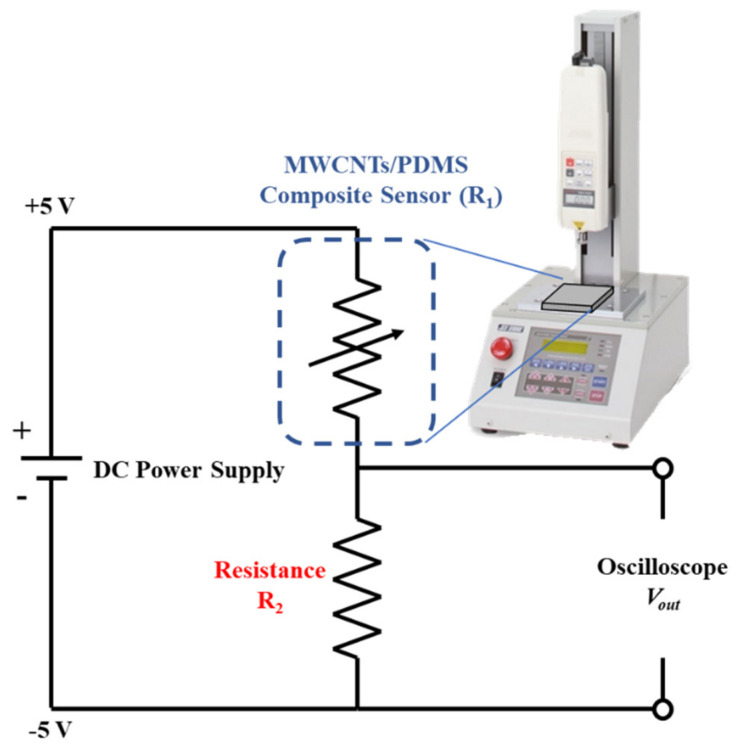
The linear conversion system architecture for signal output.

The architecture of the linear conversion system is schematically illustrated in Figure 7, and the complete experimental setup includes the sensor, static normal force test platform, power supply, voltage divider circuit, and oscilloscope.

**Figure 8:**
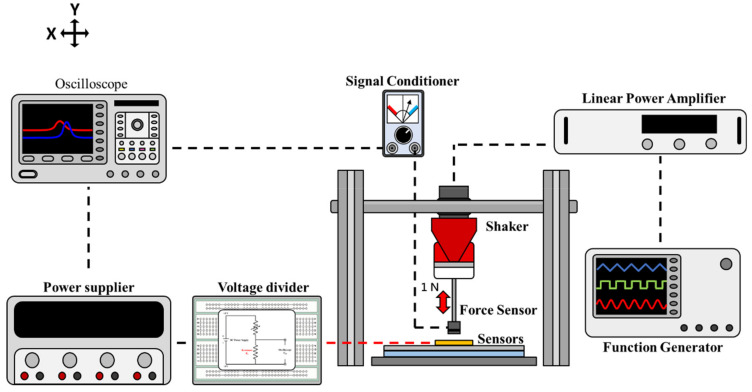
Schematic of the dynamic normal force measurement platform.

Dynamic Normal Force Measurement Platform–The dynamic normal force measurement platform is schematically illustrated in Figure 8. The acrylic disk attached to the surface of the force gauge was used to apply a normal dynamic force on the sensor surface, and the resulting pressure–corresponding voltage output characteristics of the sensor were measured. The function arbitrary waveform generator inputs sine waves of different frequencies with a 500 mV_pp_ amplitude to generate periodical motion. To test reliability, a fixed dynamic normal force of 1 N (20 kPa) was applied to the sensor surface 10 times, and the voltage output signal was displayed on the CH2 channel of the oscilloscope. The signal of the force gauge was displayed through CH1, and the response delay time can be calculated using the time difference between the waveform outputs of CH1 and CH2.

**Figure 9:**
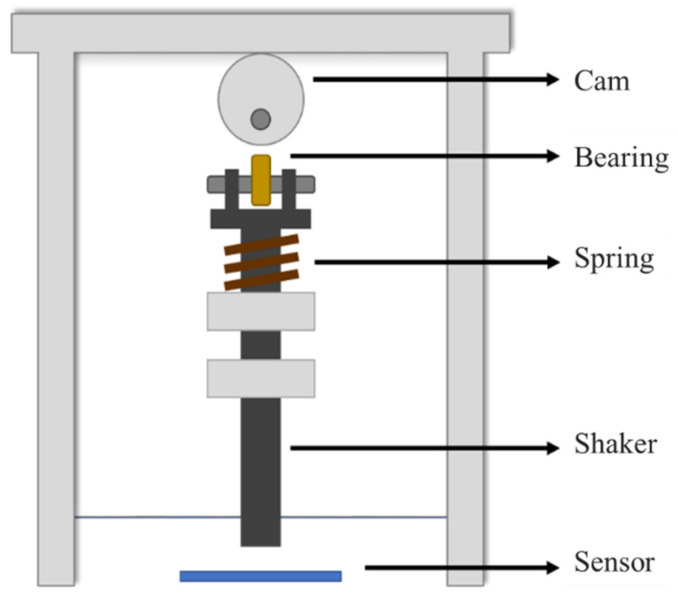
Self-made miniaturized dynamic force platform for analyzing the effects of environmental temperature on the tactile sensing response.

Temperature Measurement Platform–To measure sensor reliability as a function of environmental temperature, this study uses the same normal force of 1 N and a frequency of 1 Hz to perform dynamic testing in a temperature range of −5 °C to 50 °C. A low variation in sensor response as a function of changing temperature is ideal for force sensing applications. Since the conventional dynamic force measurement platform is too large to be placed in a temperature– and humidity–controlled chamber, we have developed a small–scale oscillating platform that can fit inside the chamber, as schematically illustrated in Figure 9. By squeezing the oscillator, a normal dynamic force was applied to the sensor whose periodic frequency was adjusted by controlling the motor speed. Finally, the variation in the sensor output signal as a function of temperature change was measured using an oscilloscope.

## 3. Results and Discussion

The tactile sensor response was analyzed under normal force loading conditions as discussed in the previous section. This section will derive a mathematical model that predicts the piezoresistive response using electron tunneling in quantum mechanics and stress-strain relationships in material mechanics. Additionally, it will discuss the observed tactile sensing response using varying MWCNT doping ratios and different sensing layer grid microstructure dimensions. Finally, the reliability of the proposed sensor was analyzed and the performance compared with commercial sensors to prove feasibility for practical applications.

### 3.1. Scanning Electron Microscopy Analysis

**Figure 10:**
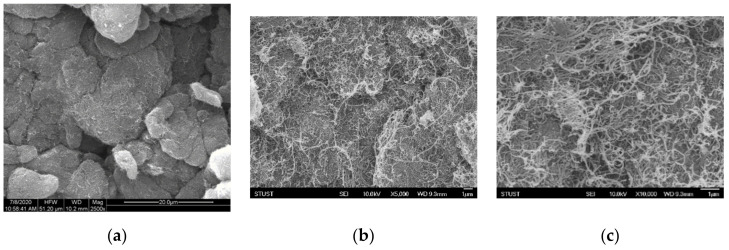
Scanning electron microscopy (SEM) images of the original MWCNT at magnifications of (**a**) 2500×, (**b**) 5000×, and (**c**) 10,000×.

**Figure 11:**
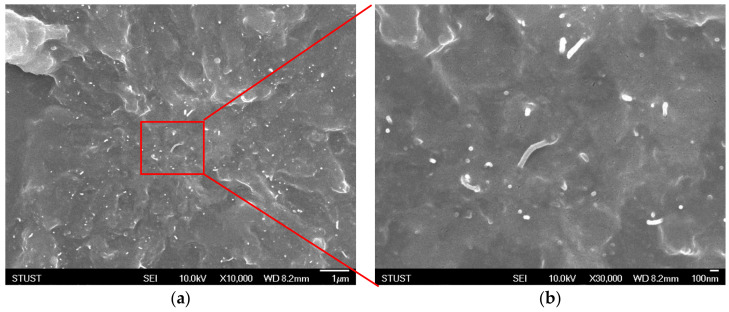
Cross–sectional SEM images of the THF–dispersed MWCNT/PDMS composite at magnifications of (**a**) 10,000× and (**b**) 30,000×.

Among the different allotropes of carbon including graphene, fullerenes, and graphite, MWCNT was chosen as the conductive filler material to fabricate the composite due to its high aspect ratio, which improves percolation effects and the resulting conductivity. The doping concentration and dispersion of MWCNT in PDMS have a great influence on the electrical performance of the resulting composite. The scanning electron microscopy (SEM) image of the original MWCNT powders is shown in Figure 10, and the agglomeration of carbon tubes can be clearly observed. Organic solvents that promote dispersion of MWCNT include toluene, THF, chloroform, and dimethylformamide (DMF) [2,3]. We have chosen THF as the organic solvent since it demonstrates improved dispersion of MWCNT and does not affect the curing and molding of PDMS. The cross–sectional SEM image of the THF-dispersed MWCNT/PDMS composite is shown in Figure 11. It was observed that MWCNTs were well dispersed in PDMS, thus confirming that the use of THF as a solvent combined with the use of ultrasonication can effectively improve the dispersion of MWCNTs in the PDMS matrix. The white dots exposed in the cross–sectional SEM image correspond to the dispersed MWCNTs, which have a length of about 10–30 um.

### 3.2. Sensor Response Characteristics

#### 3.2.1. Different MWCNT Doping Ratios

**Figure 12:**
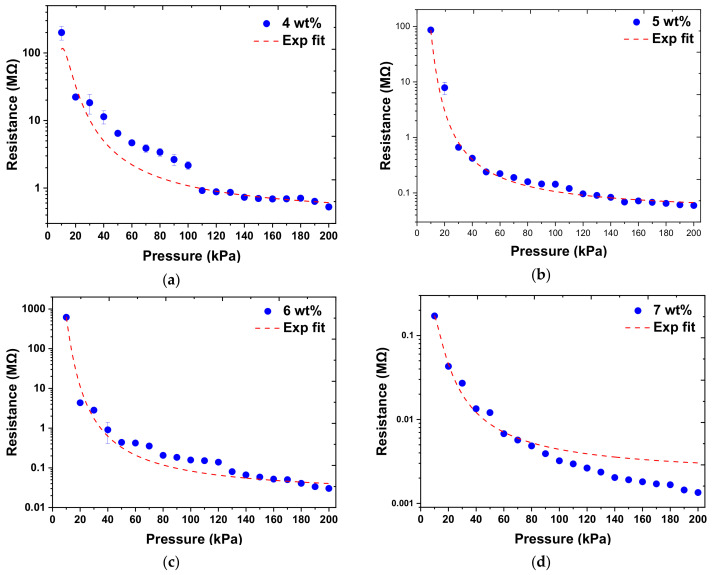

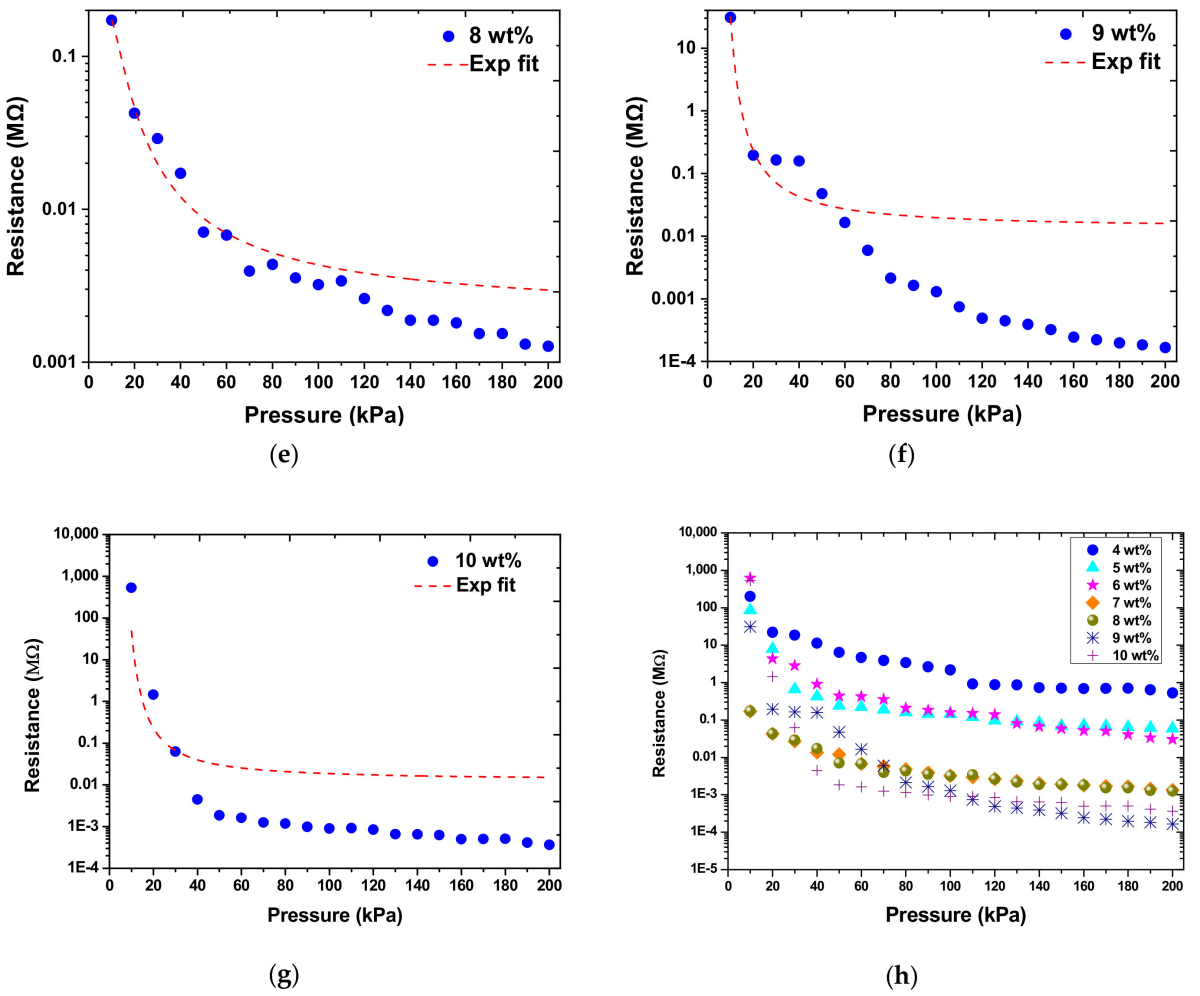
The resistance output as function of applied pressure for MWCNT doping ratios of (**a**) 4 wt.%, (**b**) 5 wt.%, (**c**) 6 wt.%, (**d**) 7 wt.%, (**e**) 8 wt.%, (**f**) 9 wt.%, (**g**) 10 wt.%, and (**h**) 4–10 wt.%.

The piezoresistive sensor response observed for normal–applied pressures in the range of 0–200 kPa was measured for different MWCNT doping ratios of 1–10 wt.%, as shown in Figure 12. When the carbon nanotubes are in contact with each other or the distance between them is very close, electrons can pass through the energy barrier via the quantum tunneling effect. For very low doping concentrations, the resistivity of the composite material was very high since the number of MWCNT was not enough to form an effective conductive network in the composite material, thereby inhibiting the current flow through it. Therefore, the composite material was in an insulating state for an MWCNT doping concentration below 3 wt.%. Although a 4 wt.% MWCNT doping concentration enables a sparse conductive network to form in the PDMS, the carbon nanotube content of the composite material was still low, and the observed resistance was high. As the doping ratio of MWCNT increases, the polymeric distance between adjacent MWCNT decreases, and a conductive path can be formed. This results in a decrease in the resistance, which is inversely proportional to the doping ratio. As the ratio of carbon tube doping increases to 10 wt.%, the MWCNT/PDMS composite material becomes highly viscous and difficult to flow, which causes difficulties in sensor manufacturing and results in a highly brittle composite. Based on these observations, 4–10 wt.% doping concentration was used for sensor response characterization. The output characteristic curve of the normal force corresponding to the resistance of the sensor with different MWCNT doping ratios. It was observed that the output resistance was inversely proportional to the applied force in the range of 0.5–10 N (10–200 kPa), thus demonstrating that the MWCNT/PDMS nanocomposite exhibits a piezoresistive response.

Several factors can affect the resistance change as a function of the applied pressure of the composite containing a conductive filler in a polymeric matrix [23,24]. This includes the filler intrinsic resistance, which changes because it is elastically deformed under pressure. However, the factor that primarily contributes to the resistance change is the polymeric thickness or distance between adjacent nanotubes, which can change considerably under applied loads. This compressive loading can decrease the polymeric gap where electrical tunneling occurs and thus result in a decrease in overall resistance. Furthermore, the percolation theory can also be applied to explain the electrical conductivity of composites containing conductive fillers such as MWCNT in an insulating matrix [25,26,27]. When the concentration of the nanotubes increases beyond a critical point, which is referred to as the percolation threshold, the composite undergoes a transition from an insulator to a conductor of electrical charge.

According to the literature, the main reasons for the piezoresistive mechanism in various carbon nanotubes (CNTs) strain sensors were (1) the tunneling effect caused by the change in the distance between adjacent CNTs, (2) the amount of contact between CNTs, or (3) the deformation of CNTs [4,5]. In the first mechanism, the tunneling resistance between two adjacent MWCNTs has an exponential relationship with the distance between the two adjacent MWCNTs, and the relationship is shown in Equation (2), where *ρ_c_* is the resistivity, *a*, *b*_1_ and *b*_2_ are constants, and X is the concentration of MWCNT. The second and third mechanisms were usually linear relationship curves, while our experimental results demonstrate an exponential relationship. Therefore, it could be concluded that the tunneling effect could be the main mechanism affecting the sensor response. After using the exponential function of Equation (2) to fit the data, the correlation coefficient can be obtained, as shown in Table 3. Curve–fitting data showed poor correlation at high doping concentrations, and it was speculated that MWCNTs in PDMS were already in contact with each other, and the distance between two carbon nanotubes was not significantly reduced under applied normal force since the composite was already in a conductive state.

(2) $$\rho_{c}=ae^{\frac{b_{1}}{X}+\frac{b_{2}}{X^{2}}}$$

It is interesting to point out that the composite piezoresistivity does not linearly increase with increasing MWCNT doping concentration. At low MWCNT concentrations, the thickness of the insulating polymer matrix is large, and electrical tunneling is low. With increasing concentrations, this thickness decreases and consequently improves electron tunneling. However, at even higher concentrations, the MWCNT network stabilizes and is hard to change under applied loading. Additionally, the agglomeration of the MWCNTs at high concentrations can render the composite brittle and result in lower efficiency of load transfer from the PDMS matrix to the CNT. Furthermore, it was also observed that the piezoresistive response of polymer nanocomposites was higher for samples with low CNT content (just above the percolation threshold), compared to highly conductive composites with high CNT content [28]. Thus, an optimized MWCNT concentration is essential to achieve improved detection sensitivities in the required pressure range.

#### 3.2.2. Optimizing the Doping Ratio of MWCNT

**Figure 13:**
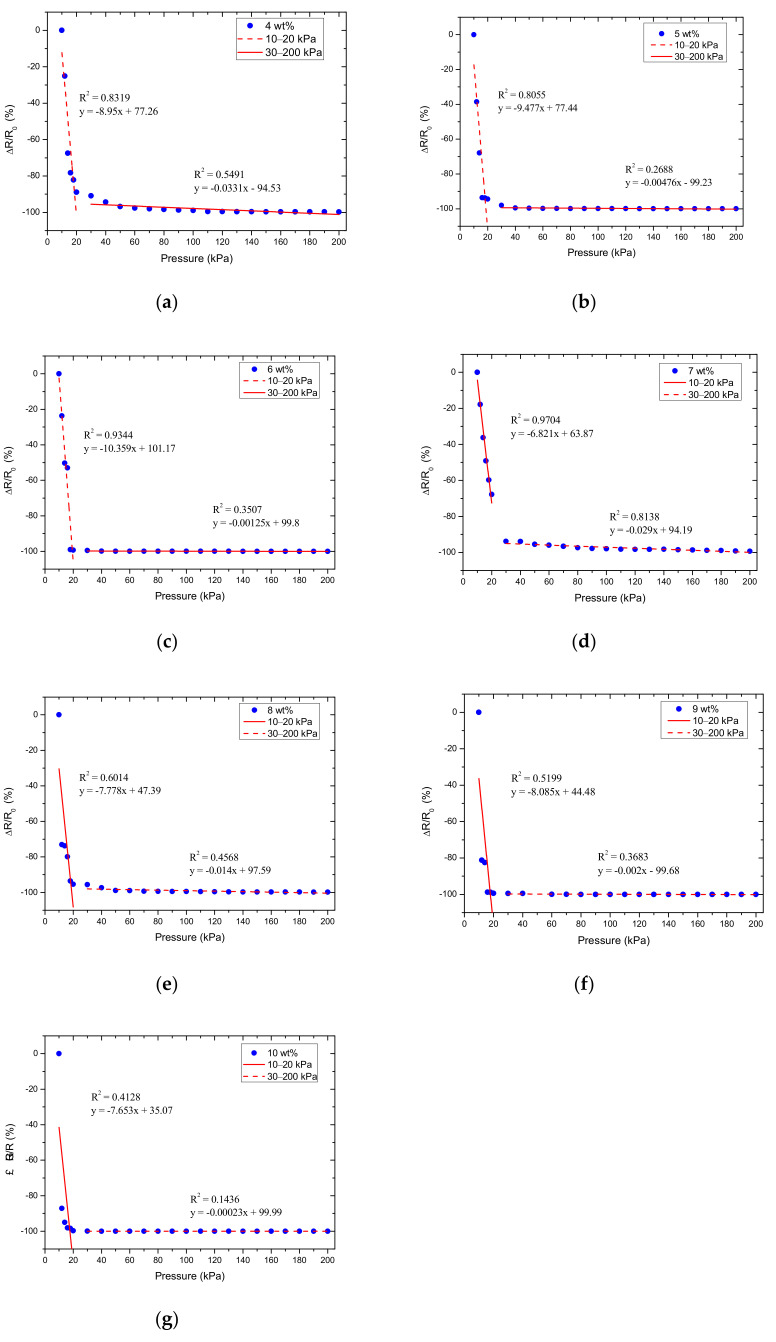
The relative resistance change as a function of applied pressure for MWCNT doping ratios of (**a**) 4 wt.%, (**b**) 5 wt.%, (**c**) 6 wt.%, (**d**) 7 wt.%, (**e**) 8 wt.%, (**f**) 9 wt.%, and (**g**) 10 wt.%.

Based on the results obtained in the previous section, we calculated the relative resistance change $\frac{\Delta R}{R_{0}}$, which was used as the Y variable, and the normal-applied pressure as the X variable to make a pressure-corresponding resistance change characteristic curve, as shown in Figure 13. The resistance of the sensor approaches infinity when there is no load. Subsequently, we used the first normal force that can be measured as the initial force F_0_, and the resulting resistance was the initial resistance *R*_0_. The resistance under a normal force was defined as *R_C_* and *R_c_* − *R*_0_ equated to Δ*R*.

**Figure 14:**
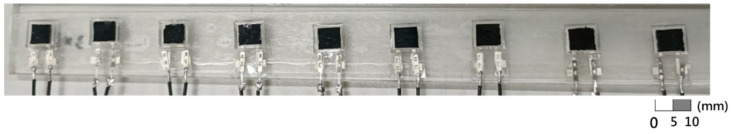
Batch production of the 7 wt.% MWCNT doped sensors.

The optimization standard in this study was to prioritize the selection of sensors with the regression curve correlation coefficient value (R) of greater than 0.90 and closer to 1 and then observe the slope of the characteristic curve that relates to the sensitivity of the sensor. A larger slope results in higher sensitivity and improved resolution. The R–value also represents the quality of linearity and can also be used to pick out a sensor with good linearity. According to the measurement results, sensors with different MWCNT doping ratios had two sensitivity ranges, namely, in the pressure range of 10–20 kPa and 30–200 kPa. The results of linear regression analysis for these two intervals under different doping ratios of MWCNT are shown in Table 4. It was observed that the 7 wt.% sensor has the most stable performance at 10–200 kPa and an image of these 7 wt.% sensors produced in a batch is shown in Figure 14.

#### 3.2.3. Voltage Output of the Sensor

**Figure 15:**
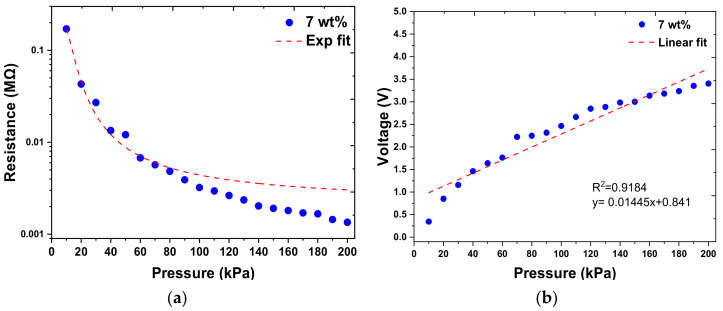
Pressure vs. resistance/voltage output for the sensors made with 7 wt.% MWCNT doping ratio. (**a**) Pressure vs. resistance response and (**b**) pressure vs. voltage response.

In this study, we can simply obtain the voltage output signal of the sensor through the circuit system, thus making it suitable for a wider range of applications. Since the pressure sensing range of our sensor is 10–200 kPa, the impedance matching of the voltage divider circuit is focused on half of the pressure sensing range, i.e., 100 kPa. According to Equation (1), the output voltage (Vout) range is 0–5 V when Vin is 5 V. Therefore, when the pressure is 100 kPa (half of the sensing range), the impedance matching resistor R2 will be selected to produce a Vout of 2.5 V (half of the output voltage range), resulting in R2 equal to R1. For example, in Figure 15a, the measured sensor impedance R1 is 3.1 kΩ at 100 kPa, and the impedance matching resistor R2 is selected with 3.1 kΩ resistance. As shown in Figure 15b, when R2 is selected as a 3.1 kΩ resistor, the measured output voltages completely fit Equation (1). When using the voltage divider circuit, the pressure–corresponding resistance response can be converted to the pressure corresponding voltage response. As shown in Figure 15b, we can use a linear fitting curve to analyze the relation between the pressure and the measured output voltage. Equation (3) was used to perform linear regression analysis on the pressure–to–voltage output characteristic curve, where the correlation coefficient R^2^ was 0.9184; a was 0.01445 kPa^−1^, which was the slope of the curve and corresponds to the sensitivity of the sensor, while b was 0.841.

(3) y=ax+b

#### 3.2.4. Different Grid Microstructures

The three parameters used for the grid structure design were line width, line spacing, and thickness, and the five levels used were 0.5, 1, 1.5, 2, and 2.5 mm. Experiments 1 to 5 used line spacing as a variable, experiments 6 to 10 used thickness as a variable, and experiments 11 to 15 used line width as a variable. The grid structure design parameters are shown in Table 5. Experiments 2, 7, and 12 were designed with the same parameters in which the line width, line spacing, and thickness were 1 mm. The sensor’s response in these experiments was measured and analyzed.

While the doping concentration of MWCNT is correlated with the resistance, the force will also cause the distance between the two carbon tubes to become smaller, which in turn will also affect the resistance change. Force is an independent variable that affects the piezoresistive response of the composite and also results in variations in the conductive network configuration near the percolation threshold. An increase in the normal applied force can result in a decrease in the polymeric gap between the MWCNT. This increases the local concentration of the MWCNT and consequently results in improved electrical tunneling. In other words, the MWCNT concentration is inversely proportional to the distance between two adjacent CNTs, which decreases as the applied force increases [19]. Thus, the applied force can be considered to be proportional to the MWCNT concentration and can affect the piezoresistive response of the composite.

**Figure 16:**
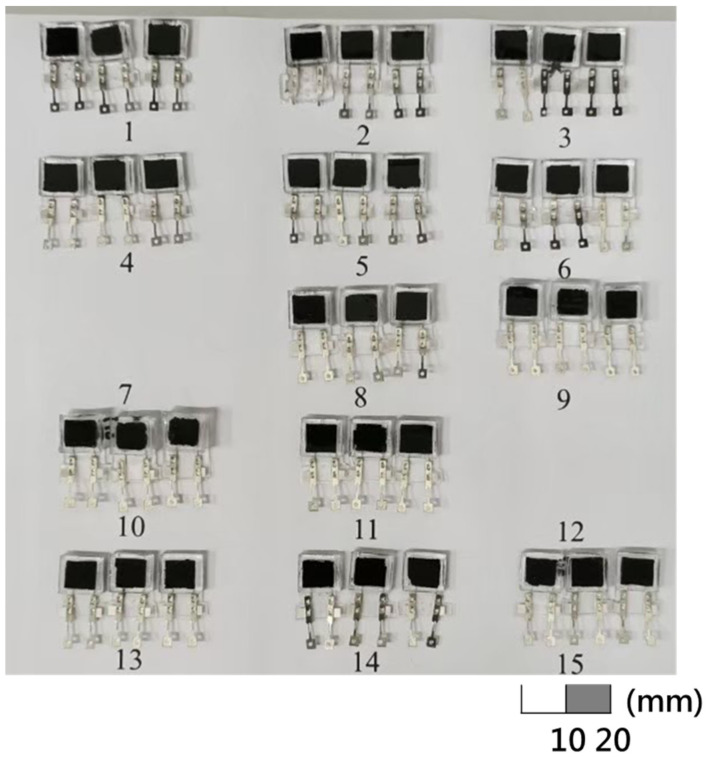
Picture of sensors in experiments 1–15 of different grid structure design.

Therefore, Equation (4) was used to predict the resistance response output and the image of the fabricated sensors for experiments 1–15 is shown in Figure 16.

(4) $$\rho_{c}=ae^{\frac{b_{1}}{F}+\frac{b_{2}}{F^{2}}}$$

**Figure 17:**
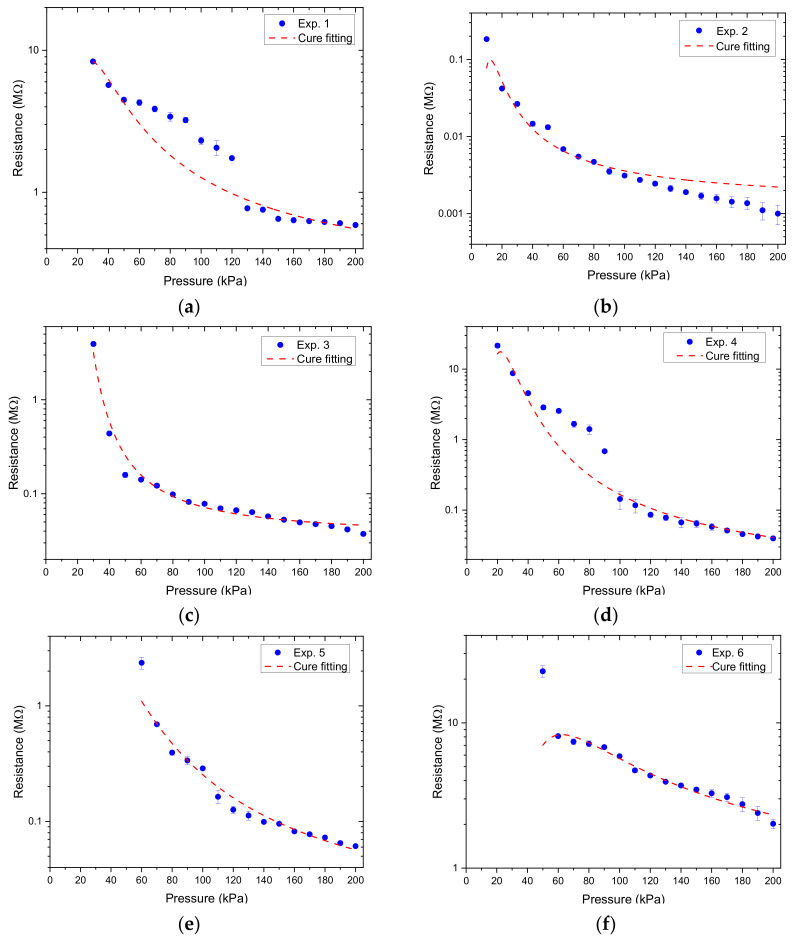

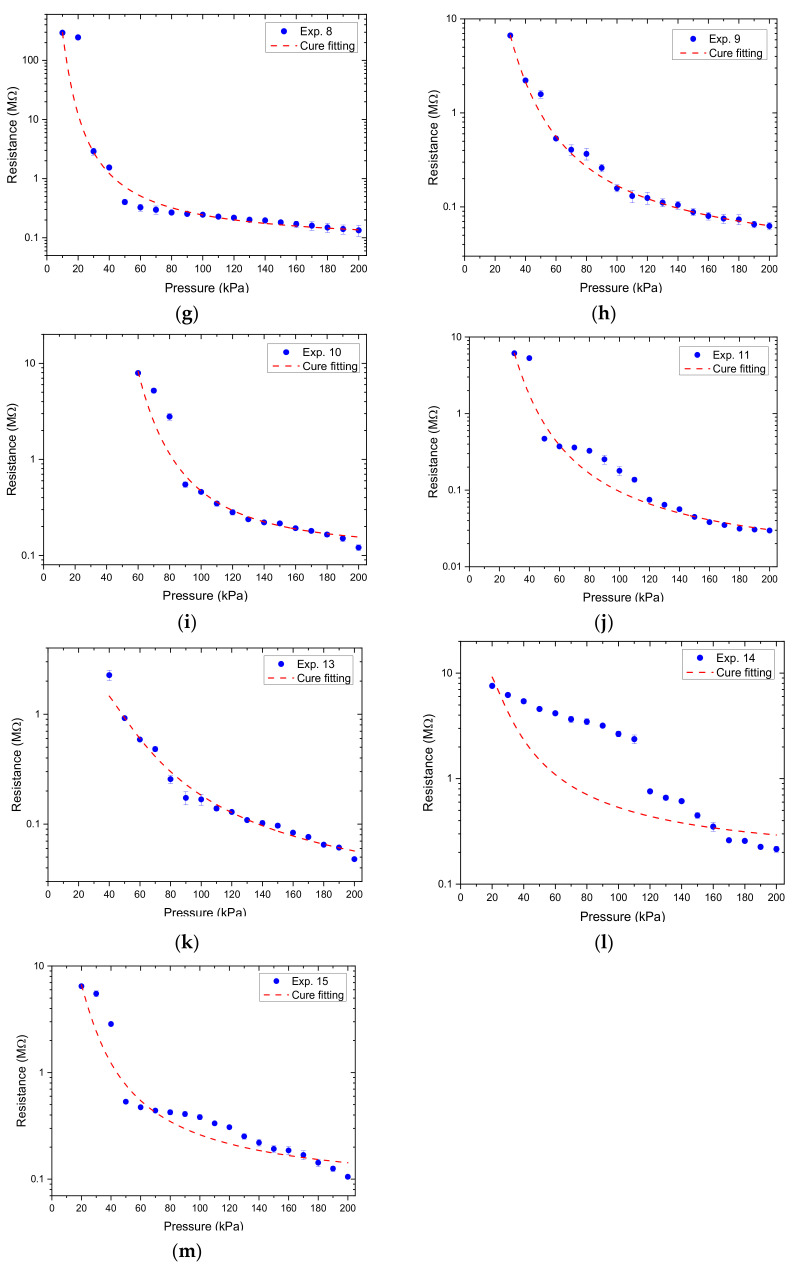
The resistance output as a function of applied pressure for different grid microstructures in which (**a**–**m**) corresponds to experiments 1–15.

**Figure 18:**
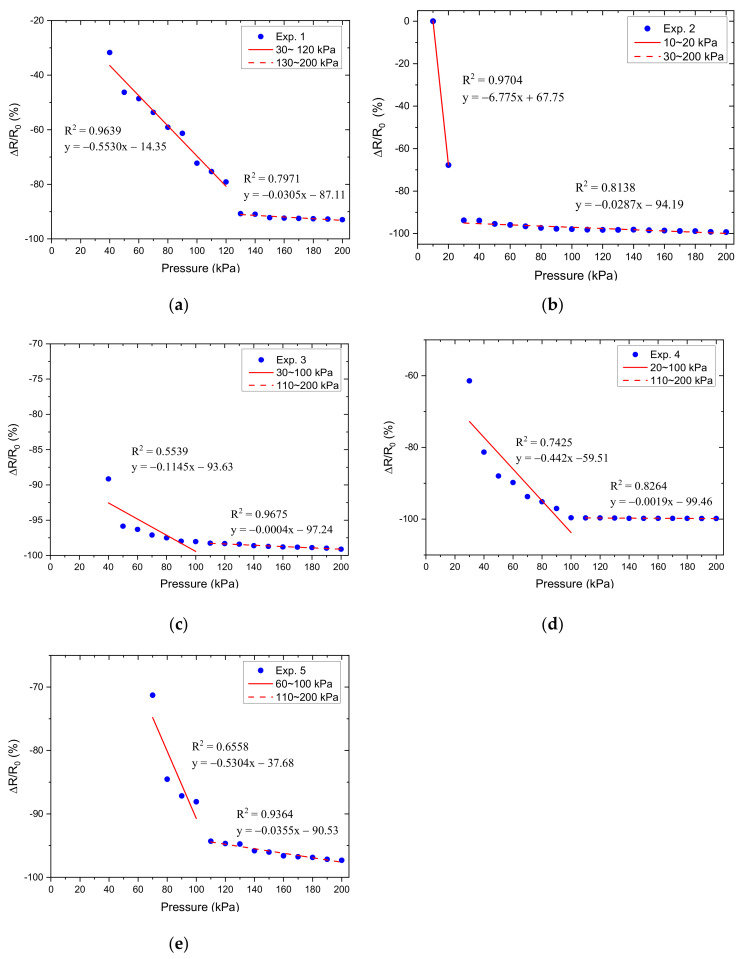
The relative resistance change as a function of applied pressure for different grid microstructures in which (**a**–**e**) corresponds to experiments 1–5.

**Figure 19:**
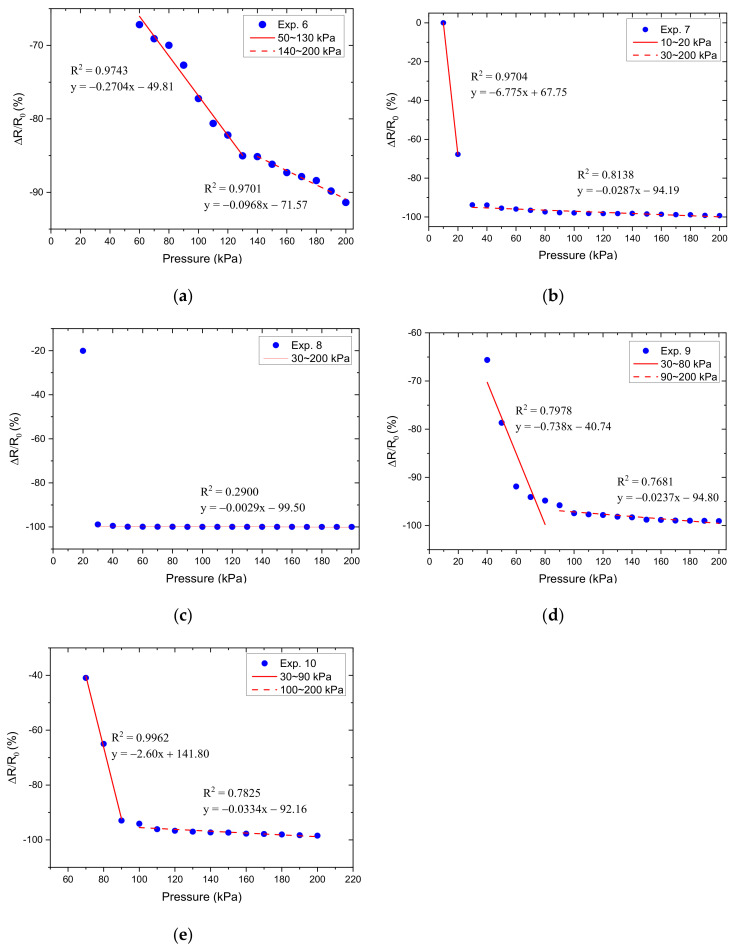
The relative resistance change as a function of applied pressure for different grid microstructures in which (**a**–**e**) corresponds to experiments 6–10.

**Figure 20:**
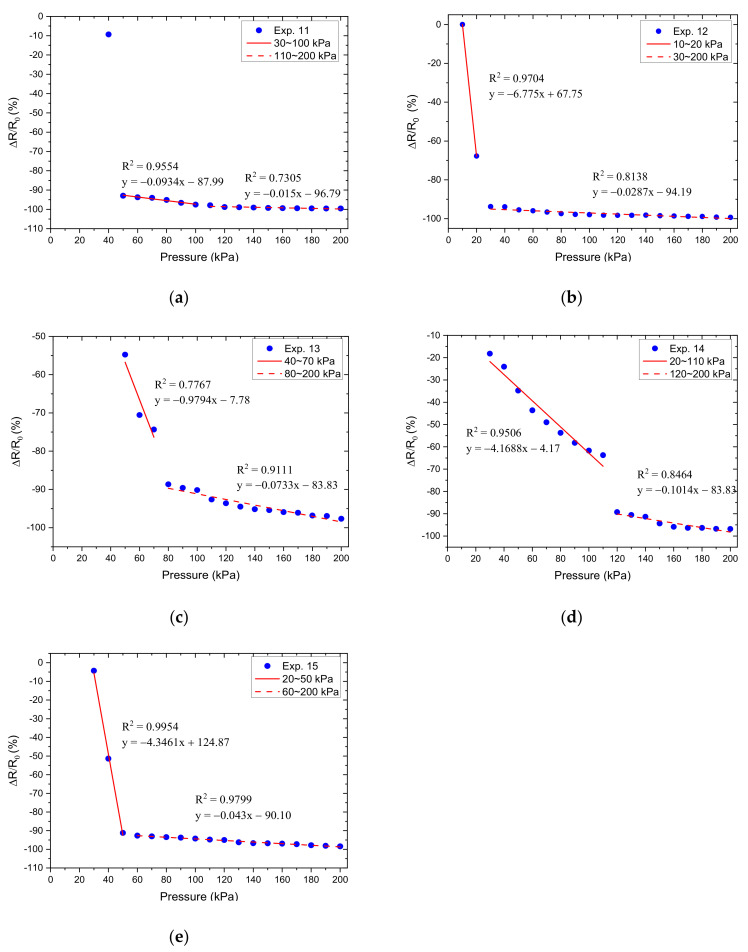
The relative resistance changes as a function of applied pressure for different grid microstructures in which (**a**–**e**) corresponds to experiments 11–15.

Based on the results obtained in Figure 17, we calculated the relative resistance change, which was used as the Y variable, and the normal-applied pressure as the X variable to make a pressure-corresponding resistance change characteristic curve, as shown in Figure 18, Figure 19 and Figure 20. Linear regression analysis was performed in which R^2^ was used to estimate the linearity. Sensors with varying grid structure parameters have different initial forces and have two distinct sensitivity values in different pressure ranges. Linear regression analysis was performed in which R^2^ was used to estimate the linearity. The results of linear regression analysis for these two ranges are shown in Table 6. For the different grid microstructures, dimensions with line width, line spacing, and thickness of 1, 1, and 0.5 mm were shown to have the most stable and improved response with a sensitivity of 0.2704 kPa^−1^ in the lower pressure range of 50–130 kPa and 0.0968 kPa^−1^ in the saturation range of 140–200 kPa.

### 3.3. Reliability Testing

#### 3.3.1. Influence of Temperature and Humidity on Sensor Response

**Figure 21:**
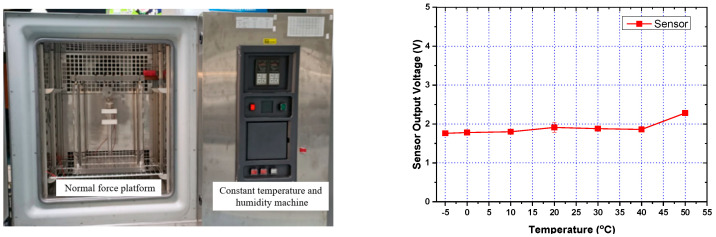
Picture of the humidity–controlled temperature chamber and the voltage output of the sensor in response to temperature changes.

**Figure 22:**
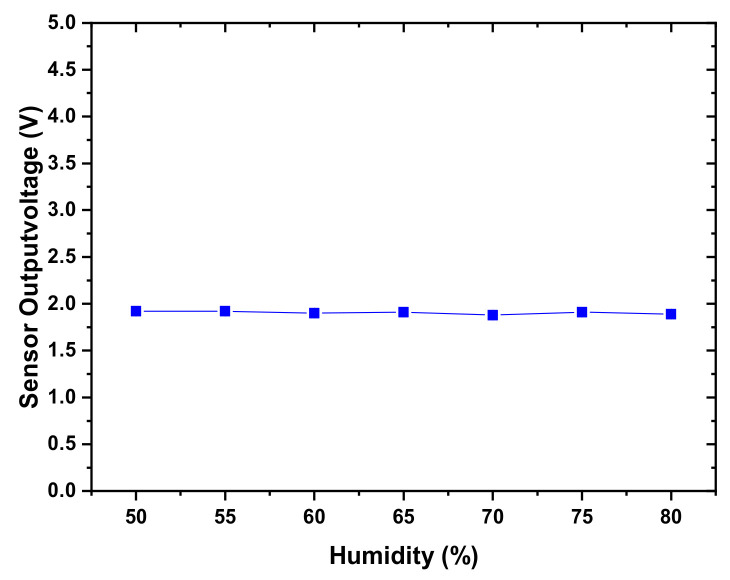
The voltage output of the sensor in response to humidity changes.

It is crucial that the ability of the sensor to perform tactile characterization is not compromised by changes in ambient temperature and humidity. We have used the sensor with an MWCNT doping concentration of 7% and grid microstructure dimensions of 1:1: 0.5 mm for which we obtained the most stable response. The applied pressure was maintained at 60 kPa, while the voltage signal output was measured in response to changes in temperature and humidity. The sensor response for temperature change in the range of 5 –50 °C is shown in Figure 21. It was observed that the signal output of the sensor was not significantly affected by temperature changes in the range of 10–40 °C, which covers the ambient environmental temperature range. Under the same applied force of 60 kPa, the voltage signal output of the sensor was analyzed under varying humidity in the range of 50–80%, as shown in Figure 22. The experimental results demonstrate that the sensor output value was minimally affected by humidity, with the largest observed variation of 2.2%. These results highlight the feasibility of the sensor to perform tactile sensing without being significantly affected by ambient temperature and humidity variations.

**Figure 23:**
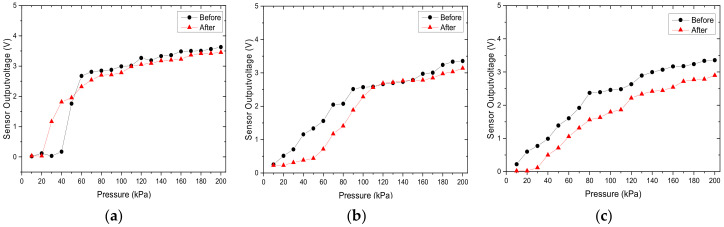
The difference in voltage output signal before and after 100 cycles of thermal testing for (**a**) sensor 1, (**b**) sensor 2, (**c**) sensor 3.

We have also performed thermal cycling tests using three sensors with an optimized MWCNT doping concentration of 7% and grid microstructure dimensions of 1:1: 0.5 mm. Using a temperature change rate of 5–15 °C per minute, a series of high– and low–temperature cycling tests were performed. The test conditions are shown in Table 7. The difference in voltage signal output of the sensor before and after cyclic thermal testing was observed, and the measurement results are shown in Figure 23. After performing 100 thermal cycles at −5 °C to 50 °C, the voltage output of the sensor decreases. This shows that if the sensor is used in a severe thermal cycling environment, it must be replaced regularly to maintain efficacy.

#### 3.3.2. Life Cycle and Stability Testing

**Figure 24:**
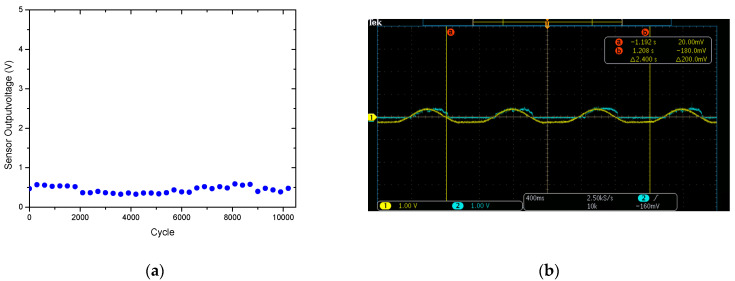
(**a**) Life cycle testing of the sensor and (**b**) the observed response delay time.

The life cycle test of the sensor is also known as its durability or service life and relates to the sensor’s ability to recover to its original performance after several cycles of deformation (loading and unloading). For practical applications, durability and repeatability are extremely important for wearable sensors or machine tools. For wearable devices, long service life is useful, especially when obtaining physiological information, which requires long–term continuous and dynamic monitoring. In this study, a dynamic normal force measurement platform was used to load/unload the sensor for 10,200 cycles at a frequency of 1 Hz under the same normal applied force of 1 N (20 kPa). It was observed that the sensor shows long–term reliability with a maximum error of 5.6% over the 10,200 dynamic cycles, as shown in Figure 24a. The results indicate that the sensor has good repeatability and the observed error over the 10,200 cycles was in the acceptable range. In Figure 24b, it can be observed that CH1 is the response of the force gauge of the dynamic normal force platform, and CH2 is the response of the sensor. From the time difference between the two response peaks, the response delay time was calculated to be 1.6 ms.

**Figure 25:**
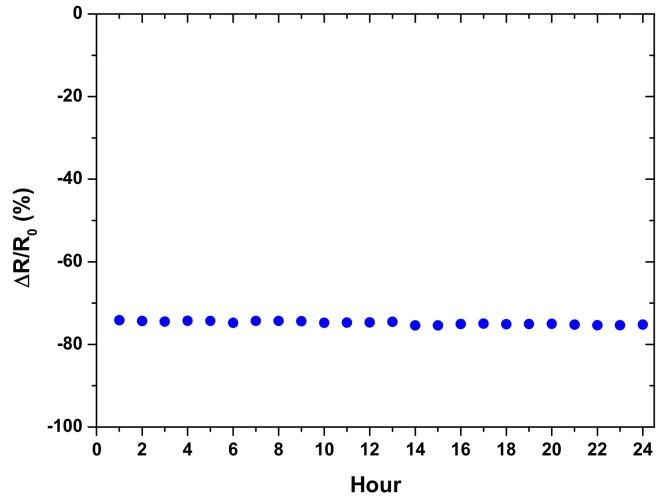
Sensor stability testing under constant loading for 24 h.

Using the same normal force of 1 N (20 kPa), the stability of the sensor under constant loading was tested. The measurement was performed every hour for 24 h, and the results are presented in Figure 25. The maximum error fluctuation was found to be 0.41%, and the resistance dropped by 0.04% after 24 h. These results indicate that the sensor shows good stability under constant loading for 24 h.

#### 3.3.3. Comparison with Commercially Available Sensors

**Figure 26:**
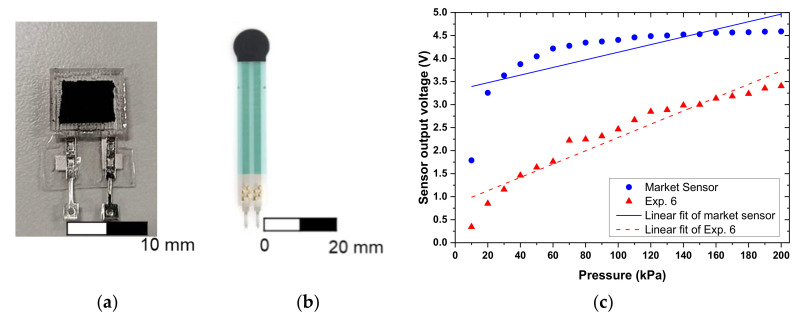
An image of the (**a**) proposed tactile sensor and (**b**) commercially available piezoresistive force sensor. (**c**) Comparison of the sensor response observed for the proposed tactile sensor and commercial sensor in the pressure range of 0–200 kPa.

At present, the printed force sensors on the market are generally made by screen printing. However, the substrate material for the commercially available sensors generally utilizes PET film, which cannot be easily deformed and does not withstand long–term deformation cycles. The sensor substrate used in this study was PDMS, which shows good performance even after multiple loading cycles. We have compared the voltage output response of the flexible force sensor proposed in this study with a commercially available sensor in the pressure range of 10–200 kPa. The commercial piezoresistive force sensor used in this study was force sensing resistors (FSR) 400 manufactured by Interlink Electronics Inc. The sensor was 7.62 mm in diameter and 0.2–1.25 mm in thickness with a force sensitivity range from 0.1–10 N. The proposed sensor used in this study has an area of about 10 × 10 mm^2^ with a thickness of about 2–3 mm and a force sensitivity range from –0.3–10 N. An image of the commercial sensor is shown in Figure 26 and the comparative results are shown in Table 8. It was observed that the error percentage of the proposed sensor and the commercially available sensor was 1.56% and 2.00%, respectively. Both the recorded errors were within the acceptable range, while the linear regression coefficient for the proposed sensor was 0.9353, which is better than the commercially available sensor with a value of 0.6995. Furthermore, the sensitivity of the proposed sensor was 0.01445 V/kPa, which is also better than the 0.00828 V/kPa observed for the commercial sensor. Based on these results, the performance of the flexible force sensor was better than that of the commercially available sensor in the pressure range of 10–200 kPa.

## 4. Conclusions

In summary, this study proposed an MWCNT/PDMS composite–based piezoresistive tactile sensor for contact force and pressure sensing. The use of THF as a solvent can enable uniform dispersion of the MWCNT before they are mixed with PDMS. Furthermore, the MWCNT/PDMS is a viscous liquid and can be stored in this form until printed using commercial techniques such as screen printing or ink–jet printing, which allows direct patterning, thus enabling low–cost manufacturing and scalability. The optimized MWCNT doping ratio was found to be about 7 wt.% with stable performance and improved sensitivity of 6.821 kPa^−1^ in the lower pressure range of 10–20 kPa and 0.029 kPa^−1^ in the saturation range of 30–200 kPa. The relationship between the electron tunneling effect and stress and strain experienced by the composite was utilized to optimize the dimensions of the grid microstructure for improving sensitivity in the low–pressure range. For the grid structure, dimensions with line width, line spacing, and thickness of 1, 1, and 0.5 mm were shown to have the most stable and improved response. The reliability test results show that the accuracy of the sensor signal response was not significantly affected by temperature and humidity. Excellent repeatability and long–term reliability were also shown within the measurement range of 10–200 kPa, thus showing promise for practical applications in wearables and robotic tactile sensing.

## Figures and Tables

**Table 1:** Parameter design for different doping ratios of multi–walled carbon nanotubes (MWCNTs).

Parameter		Value
Constant	PDMS–A (g)	2.5
	PDMS–B (g)	0.25
	THF (g)	2.5
	Grid structure (mm)	Line width:line spacing:thickness = 1:1:1
Variable	MWCNT (wt.%)	1–10

**Table 2:** Parameter design for sensing layer with different grid microstructures.

Exp.	Width	Spacing	Thickness
1.	1	0.5	1
2.	1	1	1
3.	1	1.5	1
4.	1	2	1
5.	1	2.5	1
6.	1	1	0.5
7.	1	1	1
8.	1	1	1.5
9.	1	1	2
10.	1	1	2.5
11.	0.5	1	1
12.	1	1	1
13.	1.5	1	1
14.	2	1	1
15.	2.5	1	1

**Table 3:** Correlation coefficient of exponential function for different MWCNT doping ratios.

wt.%	*a*	*b*_1_	*b*_2_
**4**	0.33118	125.14319	−667.60896
**5**	0.04174	95.26003	−191.07812
**6**	0.0192	151.27168	−474.64186
**7**	0.00205	80.11371	−358.80076
**8**	0.002	81.15095	−365.69916
**9**	0.01307	37.2995	403.70409
**10**	0.01266	33.31928	490.72404

**Table 4:** Linear regression analysis of different MWCNT wt.% sensors.

wt.%	10–20 kPa		30–200 kPa	
	Sensitivity (kPa^−1^)	R^2^	Sensitivity (kPa^−1^)	R^2^
**4**	−8.95	0.8319	−0.0331	0.5491
**5**	−9.477	0.8055	−0.00476	0.2688
**6**	−10.359	0.9344	−0.00125	0.3507
**7**	−6.821	0.9704	−0.029	0.8138
**8**	−7.778	0.6014	−0.014	0.4568
**9**	−0.014	0.5199	−0.002	0.3683
**10**	−7.653	0.4128	−0.00023	0.1436

**Table 5:** The correlation coefficient of the exponential function model for different grid microstructures.

Exp.	a	b_1_	b_2_
1	0.20616	212.29945	−3043.20202
2,7,12	0.00132	106.45368	−657.2801
3	0.03394	48.34052	2633.01675
4	0.00841	334.8633	−3666.16773
5	0.00903	402.15957	−6846.15007
6	0.56235	336.47136	−10,517.0698
8	0.07494	121.37038	−385.7058
9	0.02207	217.15394	−1372.8885
10	0.12636	−47.60155	17,890.96417
11	0.00874	12.91022	−4.61696
13	0.01395	304.97847	−4756.9036
14	0.15182	136.69884	−1093.34007
15	0.07457	134.33184	−889.65462

**Table 6:** Linear regression analysis for different grid microstructures.

Variable	Exp.	Pressure Interval (kPa)	R^2^	Sensitivity (kPa^−1^)
Spacing	1	30–120	0.9639	−0.5530
		130–200	0.7971	−0.0305
	2	−	−	−
		30–200	0.8138	−0.0287
	3	30–100	0.5539	−0.1145
		110–200	0.9675	−0.0004
	4	20–100	0.7425	−0.442
		110–200	0.8264	−0.0019
	5	60–100	0.6558	−0.5304
		110–200	0.9364	−0.0355
Thickness	6	50–130	0.9743	−0.2704
		140–200	0.9701	−0.0968
	7	−	−	−
		30–200	0.8138	−0.0287
	8	−	−	−
		30–200	0.2900	−0.0029
	9	30–80	0.7978	−0.738
		90–200	0.7681	−0.0237
	10	−	−	−
		100–200	0.7825	−0.0334
Width	11	30–100	0.9554	−0.0934
		110–200	0.7305	−0.015
	12	−	−	−
		30–200	0.8138	−0.0287
	13	−	−	−
		80–200	0.9111	−0.0733
	14	20 –110	0.9506	−4.1688
		120–200	0.8464	−0.1014
	15	−	−	−
		60–200	0.9799	−0.043

**Table 7:** Thermal cycling test condition.

Test Conditions	Parameter
Temperature range	−5–50 °C
Duration of exposure	10 min
Ramp time	10 min
Cycle time	40 min
Number of cycles	100 times

**Table 8:** Comparison of the observed error and sensitivity for the proposed sensor and the commercially available sensor.

	Proposed Sensor	Commercial Sensor
Error (%)	1.56	2
Sensitivity (V/kPa)	0.01445	0.00828

## Data Availability

Not applicable.
